# Nutrition-Related Mobile Apps in the French App Stores: Assessment of Functionality and Quality

**DOI:** 10.2196/35879

**Published:** 2022-03-14

**Authors:** Prescilla Martinon, Ina Saliasi, Denis Bourgeois, Colette Smentek, Claude Dussart, Laurie Fraticelli, Florence Carrouel

**Affiliations:** 1 Health, Systemic, Process UR 4129 Research Unit University Claude Bernard University of Lyon Lyon France

**Keywords:** mobile apps, behavior change, diet, healthy food, nutrition, prevention, mHealth, mobile health, lifestyle, French

## Abstract

**Background:**

The global burden of disease attributes 20% of deaths to poor nutrition. Although hundreds of nutrition-related mobile apps have been created, and these have been downloaded by millions of users, the effectiveness of these technologies on the adoption of healthy eating has had mixed

**Objective:**

The aim of this study was to review which nutrition-related mobile apps are currently available on the French market and assess their quality.

**Methods:**

We screened apps on the Google Play Store and the French Apple App Store, from March 10 to 17, 2021, to identify those related to nutritional health. A shortlist of 15 apps was identified, and each was assessed using the French version of the Mobile App Rating Scale: 8 dietitians and nutritionists assessed 7 apps, and the remaining apps were randomly allocated to ensure 4 assessments per app. Intraclass correlation was used to evaluate interrater agreement. Means and standard deviations of scores for each section and each item were calculated.

**Results:**

The top scores for overall quality were obtained by Yazio - *Régime et Calories* (mean 3.84, SD 0.32), FeelEat (mean 3.71, SD 0.47), and *Bonne App* (mean 3.65, SD 0.09). Engagement scores ranged from a mean of 1.95 (SD 0.5) for iEatBetter: *Journal alimentaire* to a mean of 3.85 (SD 0.44) for FeelEat. Functionality scores ranged from a mean of 2.25 (SD 0.54) for Naor to a mean of 4.25 (SD 0.46) for Yazio. Aesthetics scores ranged from a mean of 2.17 (SD 0.34) for Naor to a mean of 3.88 (SD 0.47) for Yazio. Information scores ranged from a mean of 2.38 (SD 0.60) for iEatBetter to a mean of 3.73 (SD 0.29) for Yazio. Subjective quality scores ranged from a mean of 1.13 (SD 0.25) for iEatBetter to a mean of 2.28 (SD 0.88) for *Compteur de calories* FatSecret. Specificity scores ranged from a mean of 1.38 (SD 0.64) for iEatBetter to a mean of 3.50 (SD 0.91) for FeelEat. The app-specific score was always lower than the subjective quality score, which was always lower than the quality score, which was lower than the rating from the iOS or Android app stores.

**Conclusions:**

Although prevention and information messages in apps regarding nutritional habits are not scientifically verified before marketing, we found that app quality was good. Subjective quality and specificity were associated with lower ratings. Further investigations are needed to assess whether information from these apps is consistent with recommendations and to determine the long-term impacts of these apps on users.

## Introduction

Worldwide, the burden of noncommunicable diseases continues to rise [1]. The Global Burden of Disease study [2] found that 1 in 5 deaths was due to poor diet; thus, dietary factors were responsible for 11 million deaths per year, which was more than those from any other risk factor included in the study. Several forms of malnutrition, including obesity and undernutrition, can coexist in the same population and have a significant impact on health systems. Primary health care services and lifestyle behavior improvement based on education and behavior change have great potential to decrease the global burden of noncommunicable diseases, improve health throughout the life course, and enhance well-being [3]. Thus, counseling on healthy diets and proper nutrition are among the most important nutritional interventions for promotion, prevention, treatment, and rehabilitation [4].

Mobile health is defined by the World Health Organization’s Global Observatory for eHealth as “medical and public health practice supported by mobile devices, such as mobile phones, patient monitoring devices, personal digital assistants, and other wireless devices [5].” In recent years, the number of web-based mobile health apps has increased exponentially. Currently, there are more than 325,000 mobile health apps available on major app stores. These apps are in addition to web-based health apps available on other platforms such as websites, PC software, and game consoles [6].

Furthermore, the number of apps for improving nutrition and fitness continues to grow [7]. Hundreds of nutrition-related mobile apps have been created and downloaded by millions of users over the past few years [8]. The fact that some of these apps have been downloaded numerous times indicates that people want to monitor and control their diet [9]. Access to these mobile health apps is primarily via smartphones [10]. However, it has been shown that web and mobile technologies related to nutrition have a greater impact if combined with personalized advice from a dietitian [11]. Although other prevention approaches are required, the development of effective and equitable nutrition programs is a prerequisite [12]. Since the number of apps is growing exponentially every year, it is essential to update them regularly [13]. The main industry-wide challenge is to provide credible evidence for these apps [14]. To date, little usability testing of these apps has been conducted [6]. Only a small number of English-language digital health apps have reported their usability evaluation results [6]. Although the usefulness of technologies has been demonstrated, results on the effectiveness of technology integration on the adoption of healthy eating habits are conflicting [15]. In 2018, French was spoken in 29 countries on all continents, by approximately 300 million people; 235 million people use it daily, and 90 million people are native speakers [16]; however, no overall evaluation of French-language nutrition apps has been identified in the literature.

The aim of this study was to review which nutrition-related mobile apps were available on French App stores and to evaluate their quality.

## Methods

### Selection of the French Mobile Health Apps

Two academic researchers searched for nutritional health–related apps from March 10-17, 2021 on the French Apple App Store (for iOS) and the French Google Play Store (for Android) using the following search terms: “*nutrition*” (nutrition), “*diététique*” (dietetics), “*alimentation*” (food intake), “*régime alimentaire*” (diet), and “*manger sain*” (healthy eating). Because the use of truncation and logic operators (such as AND, OR, and NOT) were not possible in the App Store and Google Play Store, each search term was provided separately.

The 2 researchers individually eliminated duplicate apps by cross-checking name of the app and the developer before comparing their respective lists. The download pages of the remaining apps were screened, and then, apps were downloaded for in-depth screening using the inclusion criteria: (1) French language, (2) targeting adult users, (3) nutrition, diet or eating habits as subject matter, (4) self-personalized programs, and (5) free (or free for at least 14 days). Mobile health apps focusing on the following topics were excluded: sports, shopping, water alert notifications only, special diet (diabetic, baby, pregnancy, vegan, religious, abstain from eating, weight gain), recipes, apps created specifically for nutritionists’ patients, meal delivery, pollution trackers, allergy and intolerance trackers, and barcode scanners.

### Selection of a Standardized Rating Scale for Mobile Apps

We used the French version of the Mobile App Rating Scale (MARS-F). The MARS-F includes 19 objective items rated with a 5-point Likert scale that are divided into 4 sections [17-21]: the *engagement* section (5 items) evaluates if the app is fun, interesting, customizable, and interactive (eg, sends alerts, messages, reminders, feedback, or allows sharing); the *functionality* section (4 items) focuses on app operation, easy to learn, navigation, flow logic, and gestural design of the app; the *aesthetic*s section (3 items) evaluates the graphic design, the overall visual appeal, the color scheme, and the stylistic consistency; and the *information quality* section (7 items) determines if the app contains high-quality information (eg, text, feedback, measurements, and references) from a credible source. The mean scores and distributions for each section were calculated. The overall MARS-F mean score was the mean score of the engagement, functionality, aesthetics, and information quality sections. Additionally, there is a *subjective* quality section (4 items), which evaluates the user’s interest for the app, and a *specificity* section, which assesses perceived effect on the user’s knowledge, attitudes, and intentions to change as well as likelihood of changing the identified targeted behaviors (we used daily habits).

### Evaluation

#### Training the Raters for Evaluation

We asked 8 dieticians and nutritionists (Multimedia Appendix 1) to rate the apps. All raters viewed a training video in French (available upon request to the corresponding author) developed for the MARS-F [22], adapted from the English-language training video [20]. To train, all raters evaluated 2 apps that had been excluded. For this, the raters downloaded and tested each app for at least 15 minutes and fulfilled the questionnaire of MARS-F. When an individual item’s rating score differed by at least 2 points, raters discussed until consensus was reached to ensure similar understanding of the item.

#### App Selection

Among the 15 apps that were included, we randomly selected 7 apps for evaluation by all raters (*Compteur de calories* FatSecret, Yazio - *Régime et Calories*, MyFitnessPal, Macros - *Compteur de calories*, Foodvisor, Lose It! - *Compteur de calories*, and *Compteur de calories*), and the remaining 8 apps were assigned to 4 raters (Lifesum: *Compteur de calories*, Naor, iEatBetter: *Journal alimentaire*, *Le secret du poids*, *Compteur de calories* ScanFood, FeelEat, Kalipi, and *Bonne App*).

The evaluation process took place from April to May 2021. The raters independently used each app for 15 minutes, and then immediately evaluated the app using a web-based MARS-F questionnaire.

### Statistical Analysis

To evaluate the interrater reliability, intraclass correlations (2-way random, average measures, absolute agreement) [23,24] and their 95% confidence intervals were calculated for the 7 common apps (for each item, section, and overall). The mean values and standard deviations were calculated for each item and for each section. Item 19 was excluded from all analyses due to missing values.

Scatter plots were used to compare differences between the quality of the apps (for each item and for each section).

The correlation between the overall quality mean and subjective item 23 (“What is your overall star rating of the app?”) was evaluated through the Pearson correlation coefficient.

Statistical analyses were performed using Stata (version 15; StataCorp LLC) and using dplyr (version 1.0.8) and ggplot2 (version 3.3.5) packages with R software (version 4.1.1; The R Project for Statistical Computing).

## Results

### Selection of Mobile Apps

**Figure 1:**
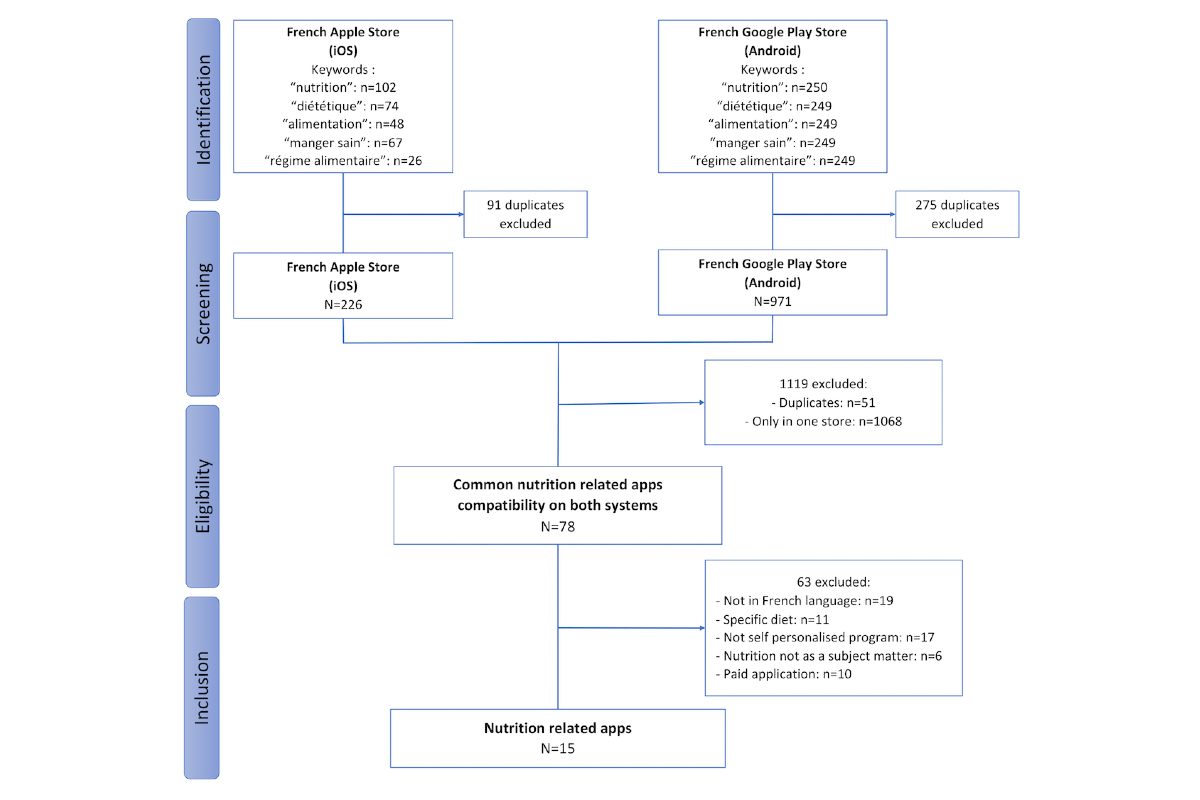
Selection flowchart.

A total of 226 apps in the Apple App Store and 971 apps in the Google Play Store were identified (Figure 1), with 78 apps available on both systems. After screening, 18 apps were preliminarily identified. After downloading, 15 apps were included.

### Characteristics of Mobile Apps

No common developer was identified for the 15 apps (Multimedia Appendix 2). Only 2 apps were fully free of charge; 1 app was free for 30 days, and the others required in-app purchases to function completely. MyFitnessPal was the most downloaded app (n=26,804 in the Apple App store and n=2,397,052 downloads in the Android App store), followed by Yazio (n=51,674 in the Apple App store and n=373,162 downloads in the Android App store) and FatSecret (n=3711 in the Apple App store and n=394,958 downloads in the Android App store).

All 15 apps targeted behavior change, goal setting, and physical health (Table 1). Most apps (10/15, 67%) focused on increasing happiness and well-being. The theoretical background and strategies used were (1) information and education, (2) monitoring and tracking, and (3) goal setting. The apps were designed for adults (15/15, 100%), young adults (15/15, 100%), adolescents (13/15, 87%), and children under 12 years (11/15, 73%). All 15 apps (100%) sent reminders, and 10 apps (10/15, 67%) required internet access to function.

**Table 1:** Characteristics of the 15 nutrition mobile apps.

Characteristic			App (n=15), n (%)^a^
**Focus or target**			
	Increase happiness or well-being	10 (67)	
	Mindfulness, meditation, or relaxation	3 (20)	
	Anxiety or stress	3 (20)	
	Behavior change	15 (100)	
	Goal setting	15 (100)	
	Relationships	1 (7)	
	Physical health	15 (100)	
**Theoretical background or strategies**			
	Assessment	10 (67)	
	Feedback	10 (67)	
	Information or education	15 (100)	
	Monitoring or tracking	15 (100)	
	Goal setting	15 (100)	
	Advice, tips, strategies, and skills training	9 (60)	
	Cognitive behavioral therapy - Behavioral (positive events)	5 (33)	
	Cognitive behavioral therapy - Cognitive (thought challenging)	5 (33)	
	Acceptance commitment therapy	4 (27)	
	Mindfulness or meditation	1 (7)	
	Relaxation	1 (7)	
	Gratitude	0 (0)	
	Strengths based	6 (40)	
	Other	0 (0)	
**Age group**			
	Children (under 12 years)	11 (73)	
	Adolescents (13-17 years)	13 (87)	
	Young adults (18-25 years)	15 (100)	
	Adults	15 (100)	
**Technical aspects of app**			
	Allows sharing (Facebook, Twitter, etc)	4 (27)	
	Has an app community	5 (33)	
	Allows password-protection	9 (60)	
	Requires log-in	2 (13)	
	Sends reminders	15 (100)	
	Needs web access to function	10 (67)	

^a^More than one could be applicable; therefore, percentages do not add to 100%.

### Reliability of the Evaluation

The reliability of the evaluations of the 7 common apps was considered good for overall quality (ICC 0.89, 95% CI 0.70-0.98) and for engagement (ICC 0.83, 95% CI 0.57-0.96), functionality (ICC 0.77, 95% CI 0.45-0.95), and aesthetics (ICC 0.83, 95% CI 0.57-0.97) sections individually. The reliability was excellent for the information quality section (ICC 0.92, 95% CI 0.78-0.98).

### Quality of the Content of the Nutrition-Related Mobile Apps

**Figure 2:**
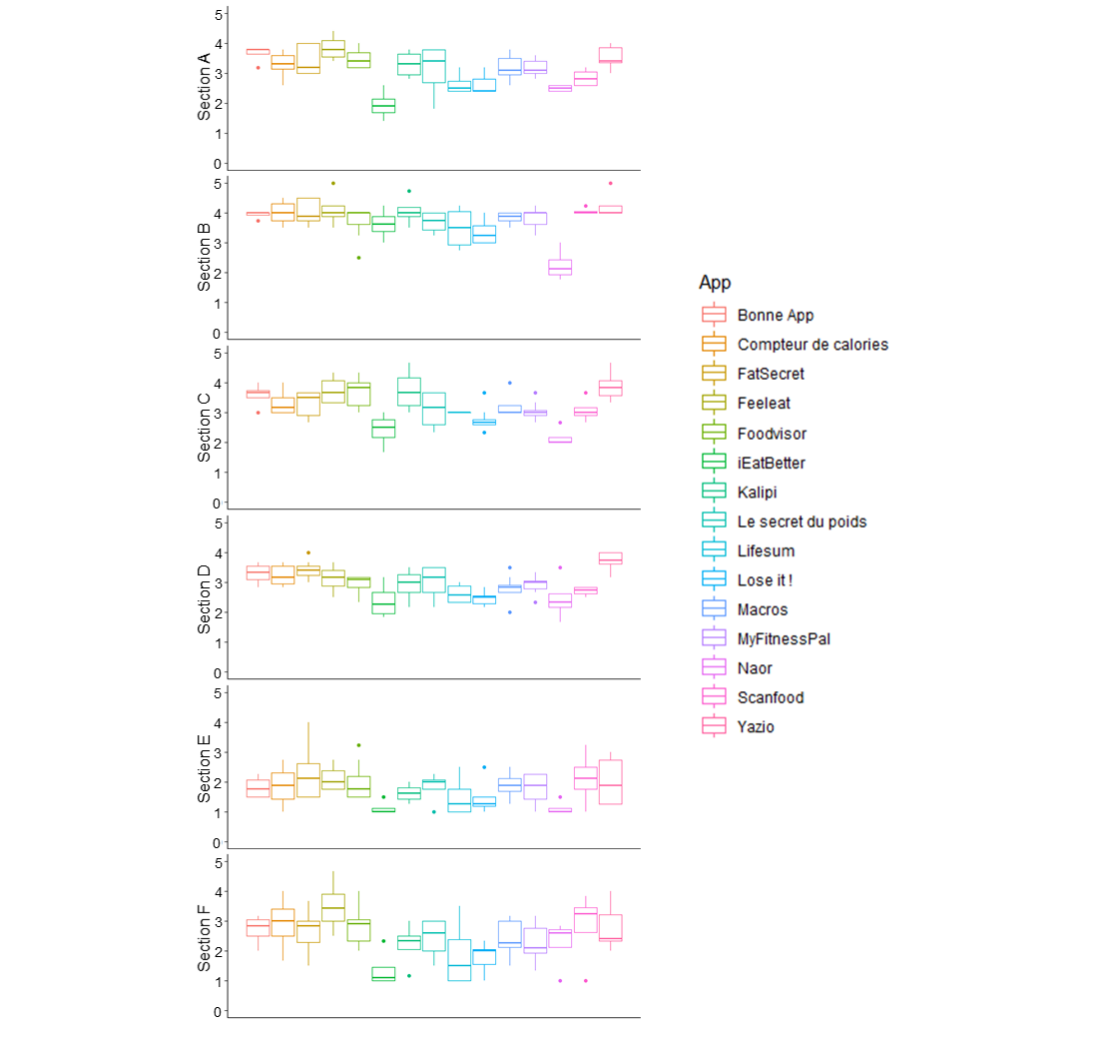
Qualitative evaluation of nutrition-related apps. Section A: Engagement; Section B: Functionality; Section C: Aesthetics; Section D: Information; Section E: Quality.

The best quality scores (Figure 2; Multimedia Appendix 3) were obtained by Yazio (mean 3.84, SD 0.32), FeelEat (mean 3.71, SD 0.47), and *Bonne App* (mean 3.65, SD 09); whereas, the worst quality scores were obtained by Naor (mean 2.34, SD 0.39), iEatBetter (mean 2.59, SD 0.40), and Lose It! (mean 2.79, SD 0.29).

The engagement scores ranged from a mean of 1.95 (SD 0.5) for iEatBetter to a mean of 3.85 (SD 0.44) for FeelEat. The functionality scores ranged from a mean of 2.25 (SD 0.54) for Naor to a mean of4.25 (SD 0.46) for Yazio. The aesthetics scores ranged from a mean of 2.17 (SD 0.34) for Naor to a mean of 3.88 (SD 0.47) for Yazio. The information quality scores ranged from a mean of 2.38 (SD 0.60) for iEatBetter to a mean of 3.73 (SD 0.29) for Yazio. For all apps, except Naor, the functionality mean score was always higher than the engagement mean score.

**Figure 3:**
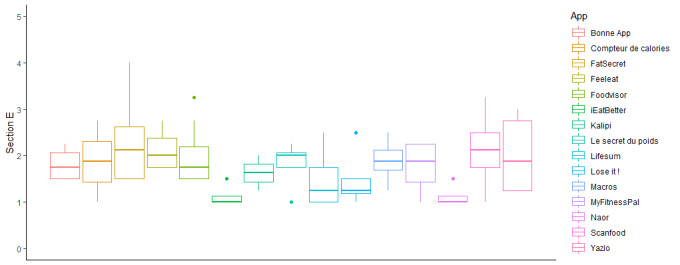
Subjective qualitative evaluation of nutrition-related apps (Section E).

The subjective quality scores (Figure 3) ranged from a mean of 1.13 (SD 0.25) for iEatBetter to a mean of 2.28 (SD 0.88) for FatSecret. The best subjective quality scores were obtained by FatSecret (mean 2.28, SD 0.88), FeelEat (mean 2.13, SD 0.48), and ScanFood (mean 2.13, SD 0.92); whereas, the worst quality scores were obtained by iEatBetter (mean 1.13, SD 0.26), Naor (mean 1.13, SD 0.25), and Lose It! (mean 1.41, SD 0.48).

### Specificity of the Content of the Nutrition-Related Mobile Apps

**Figure 4:**
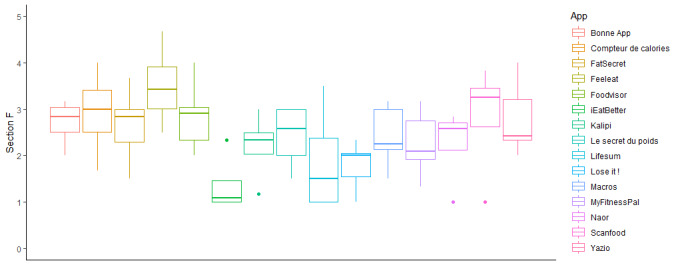
App-specific scores (Section F).

Scores for specificity of the content of the apps (Figure 4) ranged from a mean of 1.38 (SD 0.64) for iEatBetter to a mean of 3.50 (SD 0.91) for FeelEat. The best subjective quality scores were obtained by iEatBetter (mean 1.38, SD 0.64), *Compteur de calories* (mean 2.92, SD 0.79), Foodvisor (mean 2.83, SD 0.62), and ScanFood (mean 2.83, SD 1.25); whereas, the worst quality scores were obtained by Lifesum (mean 1.88, SD 1.18), Lose It! (mean 1.79, SD 0.48), and iEatBetter (mean 1.38, SD 0.64).

### Strengths and Weaknesses of Each App

The app-specific score was always lower than the subjective quality score, which was always lower than the quality score. This score was lower than the rating score from the iOS or Android app stores (Multimedia Appendix 2; Table S1 in Multimedia Appendix 3).

**Figure 5:**
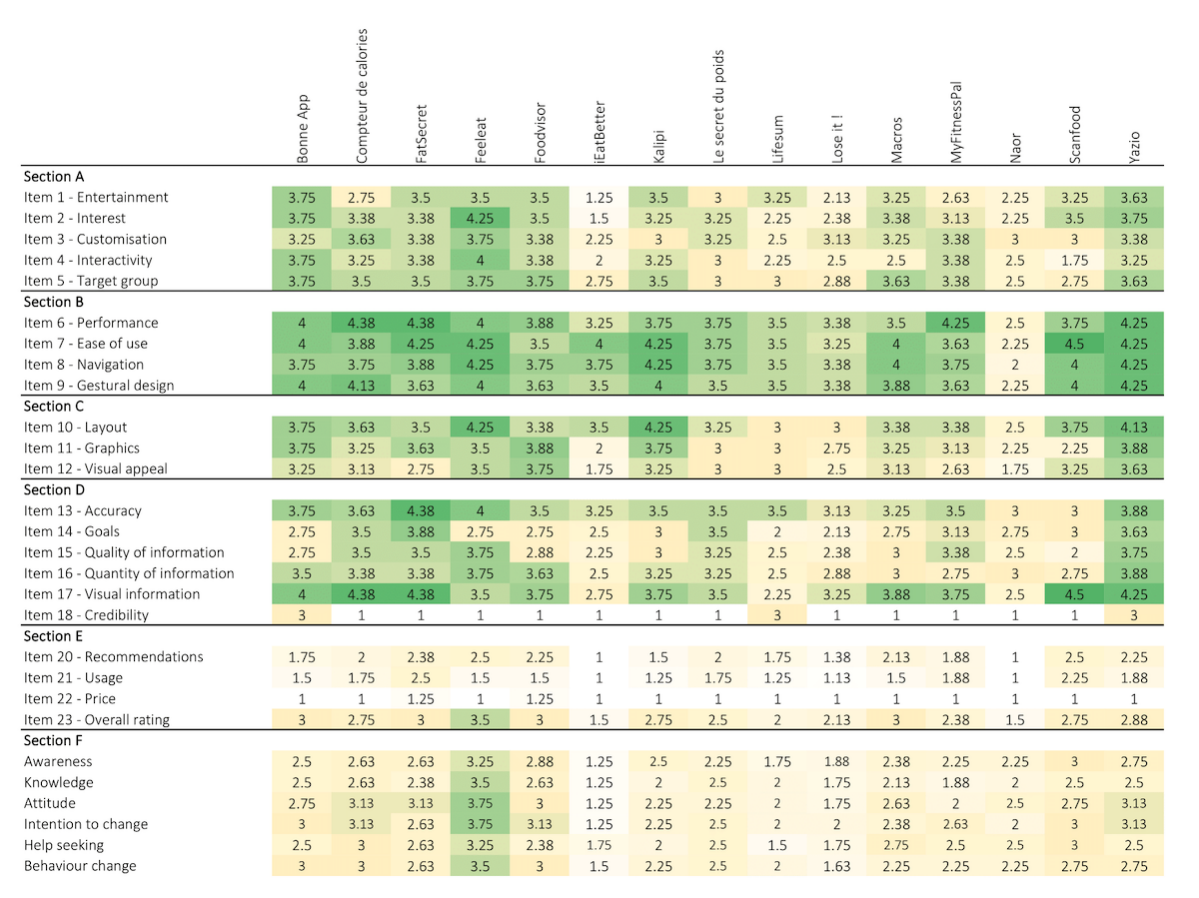
Heatmap of the average scores per item and per app, from yellow (1: worst score) to green (5: best score).

Low overall quality scores (Figure 5) were due to the information quality scores, for all apps except FatSecret, iEatBetter, Naor, and Yazio. In the information quality section, the worst score was observed for the item regarding the credibility of the app for all apps except for *Bonne App*, which obtained the worst score for goals and quality of information items, and Lifesum, which obtained the worst score for the goals item. In the subjective quality section, low scores were the result of the item indicating whether people be willing to pay for this app. The specificity scores were very close between items for the same app.

### Correlation Between MARS and Stars Ratings

The correlation between the quality mean and the subjective item 23 (“What is your overall star rating of the app?”) was considered to be good (*r*=0.67, *P*<.001) and indicated that the quality score (overall) was generally higher than that of subjective item 23.

Correlation analysis between overall MARS-F scores of the apps and their respective store ratings was limited by the availability of store ratings and the discrepancies among the number of raters. The store ratings were higher than overall MARS-F mean scores. Store ratings ranged from 3.0 (*Le secret du poids*) to 4.9 (ScanFood) for the iOS store and from 3.0 (ScanFood) to 4.7 (FatSecret) for the Android store.

## Discussion

### Principal Findings

The increasing public consciousness and high comorbidity burden related to unhealthy nutrition has highlighted the necessity of a healthy diet [25]. Nutrition behaviors can be improved by using mobile health apps, which have become very popular [26]. For diabetes [27], renal disease [28], weight loss [29], and age-related macular degeneration [30], health and nutrition professionals have used mobile health apps to monitor and encourage better lifestyle and dietary choices. The use of mobile health apps has also been found to increase adherence to dietary monitoring [29,31].

Screening of nutrition-related apps available in the French Apple App and Google Play App stores yielded 15 apps. In another study, screening of the Korean Apple app and Google Play stores yielded 29 nutrition-related apps [32]; the study [32] used 2 criteria—including only apps rated 4 stars or higher and the top 100 most reviewed apps. Another study, screening of the US Google Play Store yielded 86 apps, but the criterion for inclusion (only apps rated 4 stars or higher) was less restrictive [33].

All 15 apps targeted behavior change, physical health, and goal setting via information and education and monitoring and tracking. A previous study [34] showed that diet monitoring and education were the most frequently used functions in diet and nutrition apps [34]. All ages were targeted for 73.3% (11/15) of the apps; this finding is consistent with that from another study [33], which found that 94% of diet and nutrition apps appealed to users of all ages.

Ratings in the iOS and Android stores were higher than the MARS-F quality scores. Star ratings and user comments are valuable to users because they provide insight into the effectiveness and popularity of apps [33], but star ratings do not provide objective assessment of quality. In contrast to other studies [32,33], our study did not used star ratings as an inclusion criteria; however, for the 15 apps included (except *Bonne App* for which the number of raters or downloads was not sufficient) the star score was greater than 4.

Quality scores were greater than 2.5, except for Naor (mean 2.34, SD 0.39). Functionality was the strength for all apps, except for Naor. The high scores could be explained by the inclusion of scroll and zoom features to increase readability. The maximum score of 5 was obtained for the ease of use, navigation, and gestural design (FeelEat, iEatBetter, Yazio, and FatSecret). In contrast, Naor navigation was rated low (mean 2, SD 0.82), which could be explained by difficulty in accessing the menu, the amount of data, and the design. The weakness of all the apps, except FatSecret, iEatBetter, Naor, and Yazio, was information quality. The worst score in the information quality section was typically for credibility of the app. This corresponded to the fact that the source of information was identified, but the source’s validity or reliability was questionable (eg, commercial enterprise with vested interest). Moreover, the level of scientific evidence was difficult to evaluate. The evaluators selected “N/A The app has not been tested” in most cases; therefore, this item was not included in the statistical analysis. To the best of our knowledge, only 5 of the 15 apps (FatSecret [8,35-38], Lifesum [8,35,38], MyFitnessPal [8,35-45], Yazio [35,36,38], and Lose It! [8,35,37]) are indexed in PubMed. On the other hand, the information contained in these nutrition-related apps may have errors. For example, FatSecret, Lifesum, MyFitnessPal, and Yazio tended to underestimate total energy intake [38].

The subjective quality score were always lower than the star rating scores from the iOS and Android stores. This can be explained because the evaluations in the stores are made by all the users; whereas, in our study, dieticians or nutritionists assessed the apps using the MARS-F. Indeed, the use of user version of MARS can show different results [21].

Moreover, subjective quality scores were also lower than quality scores. This indicates that even if engagement, functionality, aesthetics, and information quality for an app were good, professionals did not think that they would use the app often in the next 12 months, and they would not be willing to pay for the app. This finding can be compared with the results of an international survey of health care professionals’ opinions on nutrition and diet apps [46]. Among 1001 health care professionals questioned, only 45.5% recommended these types apps to their patients. Surprisingly, 22.5% of people who had not yet recommended the use of these types of apps did not know of their existence. Health care professionals who have recommended apps may have used them as supplementary tools to broaden their daily practice, engage patients, enhance care, and possibly contribute to the reduction in health care costs [47]. Additionally, patients living with diseases such as diabetes or obesity may use apps for self-monitoring of their diet and physical activity [48].

Generally, raters shared a common negative opinion on the potential impact of the nutrition-related apps on the behavior change (macro micronutrients intakes), even if these apps have already demonstrated positive results in with respect to the prevention of being overweight or other chronic disease [34,49-51].

### Limitations

This study has several limitations. First, only nutrition-related apps available on both Apple and Android French stores, were included. Other stores, such as the Huawei store, the Samsung store, the Windows phone store, or BlackBerry, could have been investigated. Second, we chose to use the French version of the MARS because this scale is the most commonly used in scientific literature for mobile health app evaluation to date [52-57]. However, other scales, such as ENLIGHT or Application Quality Evaluation, which was initially specifically developed for the evaluation of mobile health app linked to nutrition purpose [58], could have been used. Third, the assessment was conducted by dieticians and nutritionists; whereas mobile health apps are intended for the general public. In further investigations, a comparison between ratings with the user version of MARS [21] and those from our study could be interesting.

### Perspectives

In a recent study [48], clinicians mentioned that nutrition apps may improve patient outcomes when compared to traditional methods of monitoring dietary and physical activity behaviors [48]. Nutrition-related apps are appealing to users, based on the high number of downloads, which supports the fact that diet intake monitoring and recommendations could be managed through these tools [8,59,60]. Thus, the findings of our study could help French users of mobile apps and professionals to select the best nutrition-related apps in terms of quality and to choose the most appropriate health literacy elements. Furthermore, when used as part of an empowerment strategy, the app must adapt to the user's chronic disease.

The implementation of new therapeutic programs that integrate mobile apps associated with follow-up with health professionals could be a key element in changing behavior. On one hand, it is important to remain vigilant with respect to the ethical issues surrounding the use of health data and the development of apps for commercial purposes. On the other hand, the discrepancy between scores obtained for the subjective quality section and for those for the specificity of the apps demonstrated that, although nutrition-related apps could be a key element in modifying the nutritional behavior of patients, for this, it is necessary to integrate the nutrition-related apps in professional practice. It would be interesting to conduct randomized clinical trials or longitudinal studies, using the 15 nutrition-related mobile apps identified in this study, to analyze nutritional behavioral modification from use of the apps and impacts on noncommunicable diseases.

## Appendix

Multimedia Appendix 1 Characteristics of the raters, the hardware, and the software used.

Multimedia Appendix 2 Descriptive and technical information of the nutrition mobile apps.

Multimedia Appendix 3 Mobile App Rating Scale (MARS) scoring.

## Abbreviations

ICC: intraclass correlation
MARS: Mobile Application Rating Scale
MARS-F: Mobile Application Rating Scale–French
