# Supplementary material for: Nutrition-Related Mobile Apps in the French App Stores: Assessment of Functionality and Quality
Source: JMIR Mhealth Uhealth. 2022 Mar 14;10(3):e35879. doi: 10.2196/35879 (PMC8961341; doi:10.2196/35879)
Supplement: Multimedia Appendix 1 [file mhealth_v10i3e35879_app1.pdf]

Multimedia Appendix 1:
Characteristics of the raters, the hardware and the software used.

	Rater 1	Rater 2	Rater 3	Rater 4	Rater 5	Rater 6	Rater 7	Rater 8
**Year of experience as nutritionist**	41	28	20	9	1	7	Student (2nd year)	11
**Affiliation**	Hospital	Health center	Hospital	Hospital	Hospital	Freelance	University	Hospital
**Scope of activity**	Undernourishment, chronic diseases, CVD*	Healthy patients, obesity, CVD*, diabetics, bowel diseases, eating disorders	Undernourishment, chronic diseases, CVD*, dialysis	Eating disorders, bariatric surgery, dialysis	CVD*, ENT**, oncology	Healthy patients, obesity, CVD*, diabetics, bowel diseases, eating disorders	NA	Research, healthy subjects
**Hardware (phone)**	HONOR	IPhone XR	Samsung Galaxy A40	HONOR 5C	IPhone 8	Xiaomi Redme Note8 Pro	Samsung Galaxy S8	Xiaomi Redme Note8
**Software (iOS or Android)**	Android	iOS	Android	Android	iOS	Android	Android	Android

*CVD: Cardiovascular Diseases

**ENT: Ear, Nose, and Throat diseases

NA: Not applicable
