# Supplementary material for: Nutrition-Related Mobile Apps in the French App Stores: Assessment of Functionality and Quality
Source: JMIR Mhealth Uhealth. 2022 Mar 14;10(3):e35879. doi: 10.2196/35879 (PMC8961341; doi:10.2196/35879)
Supplement: Multimedia Appendix 2 [file mhealth_v10i3e35879_app2.pdf]

Multimedia Appendix 2:
Descriptive and technical information of the nutrition mobile apps

Table 1. List of the 15 nutrition mobile apps included in the study and their scare rating in iOS and Android stores

App name	Developper	Rating in the iOS app store (Nb of raters)	Rating in the Android app store (Nb of downloads)	Paid Content
BonneApp	Institut du poids	not enough	3,9	Free during 30 days
Compteur de calories FatSecret	Fatsecret	4,7 (3 711)	4,7 (394 958)	Free with in-app purchases
FeelEat	Feelhealth	4,4 (81)	3,4 (191)	Free with in-app purchases
Foodvisor	Foodvisor	4,5 (33 495)	4,5 (5 143)	Free with in-app purchases
iEatBetter:Journal alimentaire	My Daily Bits LLC	4,5 (81)	4,3 (21 506)	Free with in-app purchases
Kalipi	Nutrielse	4,0 (17)	3,7 (37)	Free with in-app purchases
Le secret du poids	Michel Dargenio	3,9 (1 784)	4,2 (18 612)	Free
Lifesum: Compteur de calories	Lifesum AB	4,5 (24 013)	4,4 (268 081)	Free with in-app purchases
Lose It! - Compteur de calories	FitNow	4,6 (870)	4,6 (108 785)	Free with in-app purchases
Macros - Compteur de calories	Jose Manuel Alarcon	4,5 (63)	4,5 (8 666)	Free with in-app purchases
MyFitnessPal	Under Armour Inc	4,6 (26 804)	4,4 (2 397 052)	Free with in-app purchases
Naor	Naor Innov	4,3 (6)	4,5 (11)	Free
Compteur de calories Scanfood	Aliaksei Sakavets	4,9 (227)	3,0 (5)	Free with in-app purchases
Compteur de calories	Virtuagym	4,7 (1 339)	4,5 (62 614)	Free with in-app purchases
Yazio - Régime et Calories	YAZIO	4,6 (51 674)	4,6 (373 162)	Free with in-app purchases

Table 2. Brief description of the 15 nutrition mobile apps included in the study

App name	Brief description of apps
BonneApp	Management of menus according to objectives, monitoring of physio data (weight, glycemic load, acid-base balance, antioxidant protection, anti-cancer and anti-aging), food prescriptions and advice, management of shopping lists to facilitate the organization of purchases.<br>Free for patients of "Institut du poids" (VIP Status).
Compteur de calories FatSecret	Calorie counter provided by meals and estimate of needs based on weight, height, food plan and dietary goal data, monitoring of physical activity.
FeelEat	Calorie and nutrient counter according to objectives, emotions associated with these meals, contextualized food diary, therapeutic team, Tips and exercises, recipes and menus
Foodvisor	Monitoring of nutritional information: calories, proteins, carbohydrates, lipids, fibers, cholesterol, etc. Food diary, integrated barcode reader, Weight control, communication with nutritionists, physical activity monitoring
iEatBetter:Journal alimentaire	Food monitoring according to objectives, precise quantity entries.
Kalipi	Calorie and nutrient counter, weight monitoring, Food additives and toxicity, physical activity monitoring, Integrated barcode scanner, personalized recipes, special diet management (vegan diet, vegetarian diet, diabetes, cholesterol, convalescence, etc.)
Le secret du poids	Calorie counter (calorie gauge), weight tracking, coach
Lifesum: Compteur de calories	Diet program (ketogenic, fasting, paleo or sugar-free diet), logbook, nutrition guide, macronutrient and calorie counters, recipes, barcode scanner, physical activity tracking
Lose It! - Compteur de calories	Calorie counter, nutrient tracker, physical activity tracker, barcode reader, meal planning, eating habits analyzes, personalized recipes
Macros - Compteur de calories	Calorie counter, meal planner, macronutrient details, built-in barcode reader, receipees
MyFitnessPal	Calorie counter provided by meals and estimate of needs based on weight, height, food plan and dietary goal data, monitoring of sports activity
Naor	Food monitoring (diet or diet constraints), integrated barcode scan, photos of dishes, advice from nutritionists, nutrient and weight monitoring, alternative products. Special diets (fatty, little salty, little sweet, vegetarian, vegan, kosher, halal ...) avoiding allergies
Compteur de calories Scanfood	Food tracking based on weight goals, calorie and nutrition counter, integrated barcode scan
Compteur de calories	Calorie counter, mix coaching on nutrition and physical activity
Yazio - Régime et Calories	Nutritional informatio, calorie counter, hydration monitoring, physical activity monitoring, intermittent fasting, food programs.

Table 3. Targets of the 15 nutrition mobile apps included in the study

	BonneApp	FatSecret	FeelEat	Foodvisor	IEatBetter	Kalipi	le secret du poids	Lifesum	Lose it! - Compteur de	MACROS - compteur de	MyFitnessPal	Scanfood	Naor	Virtuagym	Yazio
**Focus: what the app targets (select all that apply)**															
Increase Happiness/Well-being	x	x	x					x	x	x	x		x	x	x
Mindfulness/Meditation/Relaxation	x		x											x	
Anxiety/Stress	x		x											x	
Behaviour Change	x	x	x	x	x	x	x	x	x	x	x	x	x	x	x
Goal Setting	x	x	x	x	x	x	x	x	x	x	x	x	x	x	x
Relationships										x					
Physical health	x	x	x	x	x	x	x	x	x	x	x	x	x	x	x

Table 4. Theoretical background and strategies of the 15 nutrition mobile apps included in the study.

	BonneApp	FatSecret	FeelEat	Foodvisor	IEatBetter	Kalipi	le secret du poids	Lifesum	Lose it! - Compteur de calorie	MACROS - compteur de calorie	MyFitnessPal	Scanfood	Naor	Virtuagym	Yazio
**Theoretical background/Strategies (all that apply)**															
Assessment					x	x	x	x	x	x	x		x	x	x
Feedback					x	x	x	x	x	x	x		x	x	x
Information/Education	x	x	x	x	x	x	x	x	x	x	x	x	x	x	x
Monitoring/Tracking	x	x	x	x	x	x	x	x	x	x	x	x	x	x	x
Goal setting	x	x	x	x	x	x	x	x	x	x	x	x	x	x	x
Advice /Tips /Strategies /Skills training	x	x	x	x				x		x	x			x	x
CBT - Behavioural (positive events)	x	x	x	x											x
CBT – Cognitive (thought challenging)	x	x	x	x											x
ACT - Acceptance commitment therapy	x	x	x	x											
Mindfulness/Meditation														x	
Relaxation														x	
Gratitude															
Strengths based	x	x	x	x				x	x						

Table 5. Age group of the 15 nutrition mobile apps included in the study.

	BonneApp	FatSecret	FeelEat	Foodvisor	IEatBetter	Kalipi	le secret du poids	Lifesum	Lose it! - Compteur de calorie	MACROS - compteur de calorie	MyFitnessPal	Scanfood	Naor	Virtuagym	Yazio
**Age group (all that apply)**															
Children (under 12)		x	x	x	x	x	x	x		x			x	x	x
Adolescents (13-17)		x	x	x	x	x	x	x	x	x	x		x	x	x
Young Adults (18-25)	x	x	x	x	x	x	x	x	x	x	x	x	x	x	x
Adults	x	x	x	x	x	x	x	x	x	x	x	x	x	x	x
General															
**Technical aspects of app (all that apply)**															
Allows sharing (Facebook, Twitter, etc.)	x						x	x			x				
Has an app community	x	x					x	x			x				
Allows password-protection	x	x	x		x			x		x	x		x	x	
Requires login			x					x							
Sends reminders	x	x	x	x	x	x	x	x	x	x	x		x	x	x
Needs web access to function	x	x	x			x		x	x	x		x	x		x

Table 6. Technical aspects of the 15 nutrition mobile apps included in the study.

	BonneApp	FatSecret	FeelEat	Foodvisor	IEatBetter	Kalipi	le secret du poids	Lifesum	Lose it! - Compteur de calorie	MACROS - compteur de calorie	MyFitnessPal	Scanfood	Naor	Virtuagym	Yazio
**Technical aspects of app (all that apply)**															
Allows sharing (Facebook, Twitter, etc.)	x						x	x			x				
Has an app community	x	x					x	x			x				
Allows password-protection	x	x	x		x			x		x	x		x	x	
Requires login			x					x							
Sends reminders	x	x	x	x	x	x	x	x	x	x	x		x	x	x
Needs web access to function	x	x	x			x		x	x	x		x	x		x
