# Supplementary material for: Nutrition-Related Mobile Apps in the French App Stores: Assessment of Functionality and Quality
Source: JMIR Mhealth Uhealth. 2022 Mar 14;10(3):e35879. doi: 10.2196/35879 (PMC8961341; doi:10.2196/35879)
Supplement: Multimedia Appendix 3 [file mhealth_v10i3e35879_app3.pdf]

Multimedia Appendix 3:
Mobile App Rating Scale (MARS) scoring

Table 1. Scoring by section

App Name	Data	Section A	Section B	Section C	Section D	Section ABCD	Section E	Section F
BonneApp	Mean	3,65	3,94	3,58	3,29	3,62	1,81	2,71
	SD	0.30	0.13	0.42	0.37	0.06	0.38	0.52
Compteur de calories FatSecret	Mean	3,43	4,03	3,29	3,42	3,54	2,28	2,67
	SD	0.48	0.41	0.45	0.33	0.35	0.88	0.66
FeelEat	Mean	3,85	4,13	3,75	3,13	3,71	2,13	3,50
	SD	0.44	0.63	0.50	0.50	0.50	0.48	0.91
Foodvisor	Mean	3,50	3,86	3,79	3.02	3,58	2,00	2,83
	SD	0.63	0.71	0.69	0.49	0.51	0.65	0.62
iEatBetter:Journal alimentaire	Mean	1,95	3,63	2,42	2,38	2,59	1,13	1,38
	SD	0.50	0.52	0.57	0.60	0.41	0.25	0.64
Kalipi	Mean	3,30	4,06	3,75	2,92	3,51	1,63	2,21
	SD	0.48	0.52	0.74	0.57	0.45	0.32	0.76
Le secret du poids	Mean	3,10	3,69	3,08	3,00	3,22	1,81	2,42
	SD	0.95	0.38	0.69	0.64	0.64	0.55	0.73
Lifesum: Compteur de calories	Mean	2,65	3,50	3,00	2,63	2,94	1,50	1,88
	SD	0.38	0.74	0.00	0.34	0.33	0.71	1.18
Lose It! - Compteur de calories	Mean	2,60	3,41	2,75	2,46	2,80	1,41	1,79
	SD	0.39	0.52	0.43	0.23	0.36	0.48	0.48
Macros - Compteur de calories	Mean	3,13	3,91	3,25	2,81	3,28	1,91	2,42
	SD	0.58	0.30	0.46	0.43	0.35	0.44	0.58
MyFitnessPal	Mean	3,18	3,81	3,08	2,92	3,24	1,78	2,25
	SD	0.26	0.37	0.43	0.31	0.30	0.49	0.64
Naor	Mean	2,50	2,25	2,17	2,46	2,34	1,13	2,25
	SD	0.12	0.54	0.33	0.76	0.38	0.25	0.84
Compteur de calories Scanfood	Mean	2,85	4,06	3,08	2,71	3,18	2,13	2,83
	SD	0.30	0.13	0.42	0.16	0.19	0.92	1.25
Compteur de calories	Mean	3,30	4,03	3,33	3,23	3,47	1,88	2,92
	SD	0.39	0.36	0.44	0.36	0.34	0.61	0.79
Yazio - Régime et Calories	Mean	3,53	4,25	3,88	3,73	3,84	2,00	2,79
	SD	0.37	0.46	0.47	0.29	0.33	0.74	0.75

Table 2. Scoring by items

App Name	Data	Section A					Section B				Section C			Section D						
		Item 1	Item 2	Item 3	Item 4	Item 5	Item 6	Item 7	Item 8	Item 9	Item 10	Item 11	Item 12	Item 13	Item 14	Item 15	Item 16	Item 17	Item 18	Item 19
BonneApp	Mean	3,75	3,75	3,25	3,75	3,75	4,00	4,00	3,75	4,00	3,75	3,75	3,25	3,75	2,75	2,75	3,50	4,00	3,00	NA
	SD	0,50	0,50	0,50	0,50	0,50	0,00	0,00	0,50	0,00	0,50	0,50	0,50	0,50	0,96	0,50	0,58	0,00	0,00	NA
Compteur de calories FatSecret	Mean	3,50	3,38	3,38	3,38	3,50	4,38	4,25	3,88	3,63	3,50	3,63	2,75	4,38	3,88	3,50	3,38	4,38	1,00	2,00
	SD	0,53	0,52	0,52	0,52	0,53	0,52	0,46	0,35	0,74	0,53	0,52	0,46	0,52	0,64	0,53	0,52	0,52	0,00	0,00
Feeleat	Mean	3,50	4,25	3,75	4,00	3,75	4,00	4,25	4,25	4,00	4,25	3,50	3,50	4,00	2,75	3,75	3,75	3,50	1,00	NA
	SD	0,58	0,50	0,96	0,00	0,50	0,82	0,50	0,50	0,82	0,50	0,58	0,58	0,82	0,96	0,50	0,50	0,58	0,00	NA
Foodvisor	Mean	3,50	3,50	3,38	3,38	3,75	3,88	3,50	3,75	3,63	3,38	3,88	3,75	3,50	2,75	2,88	3,63	3,75	1,00	NA
	SD	0,53	0,53	0,52	0,52	0,46	0,35	0,76	0,71	0,74	0,74	0,64	0,46	0,53	0,71	0,64	0,52	0,46	0,00	NA
iEatBetter:Journal alimentaire	Mean	1,25	1,50	2,25	2,00	2,75	3,25	4,00	3,75	3,50	3,50	2,00	1,75	3,25	2,50	2,25	2,50	2,75	1,00	NA
	SD	0,50	0,58	0,50	0,82	0,50	0,50	0,82	0,50	0,58	1,00	0,82	0,50	0,96	0,58	1,26	1,00	0,96	0,00	NA
Kalipi	Mean	3,50	3,25	3,00	3,25	3,50	3,75	4,25	4,25	4,00	4,25	3,75	3,25	3,50	3,00	3,00	3,25	3,75	1,00	NA
	SD	0,58	0,96	0,00	0,50	0,58	0,50	0,50	0,50	0,82	0,96	0,96	0,50	0,58	0,82	0,82	0,96	0,50	0,00	NA
Le secret du poids	Mean	3,00	3,25	3,25	3,00	3,00	3,75	3,75	3,75	3,50	3,25	3,00	3,00	3,50	3,50	3,25	3,25	3,50	1,00	NA
	SD	1,41	0,96	0,96	0,82	0,82	0,50	0,50	0,50	0,58	0,96	0,82	0,82	0,58	1,00	0,96	0,96	0,58	0,00	NA
Lifesum: Compteur de calories	Mean	3,25	2,25	2,50	2,25	3,00	3,50	3,50	3,50	3,50	3,00	3,00	3,00	3,50	2,00	2,50	2,50	2,25	3,00	2,00
	SD	0,50	0,50	0,58	0,50	0,00	0,58	0,58	0,58	1,29	0,00	0,00	0,00	0,58	0,00	0,58	0,58	0,50	0,00	0,00
Lose It! - Compteur de calories	Mean	2,13	2,38	3,13	2,50	2,88	3,38	3,25	3,38	3,38	3,00	2,75	2,50	3,13	2,13	2,38	2,88	3,25	1,00	NA
	SD	0,35	0,52	0,35	0,53	0,35	0,52	0,46	0,52	0,52	0,53	0,71	0,53	0,64	0,35	0,52	0,35	0,46	0,00	NA
Macros - Compteur de calories	Mean	3,25	3,38	3,25	2,50	3,63	3,50	4,00	4,00	3,88	3,38	3,25	3,13	3,25	2,75	3,00	3,00	3,88	1,00	NA
	SD	0,46	0,52	0,46	0,53	0,74	0,53	0,00	0,00	0,35	0,52	0,46	0,64	0,71	0,71	0,76	0,53	0,35	0,00	NA
MyFitnessPal	Mean	2,63	3,13	3,38	3,38	3,38	4,25	3,63	3,75	3,63	3,38	3,13	2,63	3,50	3,13	3,38	2,75	3,75	1,00	4,00
	SD	0,52	0,35	0,52	0,52	0,52	0,46	0,52	0,46	0,52	0,52	0,35	0,52	0,53	0,83	0,52	0,46	0,46	0,00	0,00
Naor	Mean	2,25	2,25	3,00	2,50	2,50	2,50	2,25	2,00	2,25	2,50	2,25	1,75	3,00	2,75	2,50	3,00	2,50	1,00	NA
	SD	0,50	0,50	0,00	0,58	0,58	1,00	0,50	0,82	0,50	0,58	0,50	0,50	0,82	0,96	1,00	0,82	1,29	0,00	NA
Compteur de calories Scanfood	Mean	3,25	3,50	3,00	1,75	2,75	3,75	4,50	4,00	4,00	3,75	2,25	3,25	3,00	3,00	2,00	2,75	4,50	1,00	NA
	SD	0,50	0,58	0,00	0,50	0,96	0,50	0,58	0,00	0,00	0,50	1,26	0,50	0,82	1,15	0,82	0,50	0,58	0,00	NA
Compteur de calories	Mean	2,75	3,38	3,63	3,25	3,50	4,38	3,88	3,75	4,13	3,63	3,25	3,13	3,63	3,50	3,50	3,38	4,38	1,00	2,00
	SD	0,46	0,52	0,52	0,46	0,76	0,52	0,35	0,46	0,64	0,52	0,46	0,64	0,52	0,53	0,53	0,52	0,52	0,00	0,00
Yazio - Régime et Calories	Mean	3,63	3,75	3,38	3,25	3,63	4,25	4,25	4,25	4,25	4,13	3,88	3,63	3,88	3,63	3,75	3,88	4,25	3,00	2,00
	SD	0,52	0,46	0,52	0,46	0,52	0,46	0,46	0,46	0,46	0,35	0,83	0,52	0,35	0,52	0,46	0,35	0,71	0,00	0,00

App Name	Data	Section E				Mobile app specificities					
		Item 20	Item 21	Item 22	Item 23	Awareness	Knowledge	Attitudes	Intention to change	Help seeking	Behaviour Change
BonneApp	Mean	1,75	1,50	1,00	3,00	2,50	2,50	2,75	3,00	2,50	3,00
	SD	0,96	0,58	0,00	0,00	0,58	0,58	0,96	0,82	0,58	0,82
Compteur de calories FatSecret	Mean	2,38	2,50	1,25	3,00	2,63	2,38	3,13	2,63	2,63	2,63
	SD	0,92	1,41	0,71	0,93	1,06	0,52	0,99	0,74	1,06	0,74
Feeleat	Mean	2,50	1,50	1,00	3,50	3,25	3,50	3,75	3,75	3,25	3,50
	SD	0,58	1,00	0,00	0,58	0,50	1,00	0,96	0,96	1,50	1,29
Foodvisor	Mean	2,25	1,50	1,25	3,00	2,88	2,63	3,00	3,13	2,38	3,00
	SD	0,71	0,93	0,71	0,76	0,83	0,74	0,93	0,64	1,06	0,76
iEatBetter:Journal alimentaire	Mean	1,00	1,00	1,00	1,50	1,25	1,25	1,25	1,25	1,75	1,50
	SD	0,00	0,00	0,00	1,00	0,50	0,50	0,50	0,50	0,96	1,00
Kalipi	Mean	1,50	1,25	1,00	2,75	2,50	2,00	2,25	2,25	2,00	2,25
	SD	0,58	0,50	0,00	0,50	0,58	0,82	0,96	0,96	0,82	0,96
Le secret du poids	Mean	2,00	1,75	1,00	2,50	2,25	2,50	2,25	2,50	2,50	2,50
	SD	0,82	0,50	0,00	1,00	0,96	1,00	0,96	0,58	0,58	0,58
Lifesum: Compteur de calories	Mean	1,75	1,25	1,00	2,00	1,75	2,00	2,00	2,00	1,50	2,00
	SD	0,96	0,50	0,00	1,41	0,96	1,41	1,41	1,41	0,58	1,41
Lose It! - Compteur de calories	Mean	1,38	1,13	1,00	2,13	1,88	1,75	1,75	2,00	1,75	1,63
	SD	0,74	0,35	0,00	0,99	0,64	0,71	0,46	0,76	0,46	0,52
Macros - Compteur de calories	Mean	2,13	1,50	1,00	3,00	2,38	2,13	2,63	2,38	2,75	2,25
	SD	0,64	0,76	0,00	0,76	0,52	0,64	0,92	0,74	0,71	1,04
MyFitnessPal	Mean	1,88	1,88	1,00	2,38	2,25	1,88	2,00	2,63	2,50	2,25
	SD	0,64	0,83	0,00	0,74	0,71	0,83	0,93	0,74	0,93	1,04
Naor	Mean	1,00	1,00	1,00	1,50	2,25	2,00	2,50	2,00	2,50	2,25
	SD	0,00	0,00	0,00	1,00	0,96	0,82	1,00	0,82	1,29	0,96
Compteur de calories Scanfood	Mean	2,50	2,25	1,00	2,75	3,00	2,50	2,75	3,00	3,00	2,75
	SD	1,00	1,89	0,00	1,26	1,41	1,29	1,26	1,41	1,41	1,26
Compteur de calories	Mean	2,00	1,75	1,00	2,75	2,63	2,63	3,13	3,13	3,00	3,00
	SD	0,93	1,04	0,00	1,04	1,06	1,19	0,83	0,99	1,07	1,07
Yazio - Régime et Calories	Mean	2,25	1,88	1,00	2,88	2,75	2,50	3,13	3,13	2,50	2,75
	SD	1,04	1,25	0,00	0,99	1,04	1,20	0,83	0,83	0,93	1,16
